# DL‐3‐n‐Butylphthalide Protects Against PrP^106^ ^−126^‐Induced Neurotoxicity Through NRF2 Signaling and OPA1/DRP1‐Mediated Mitochondrial Dynamics

**DOI:** 10.1002/cns.70948

**Published:** 2026-06-02

**Authors:** Wei Wu, Xixi Zhang, Mingyue Jiang, Ning Ma

**Affiliations:** ^1^ The Fifth Affiliated Hospital of Guangzhou Medical University Guangzhou Guangdong China; ^2^ Department of Basic Research Guangzhou National Laboratory Guangzhou Guangdong China; ^3^ Center for Neurometabolism and Regenerative Medicine Bioland Laboratory Guangzhou Guangdong China; ^4^ The Affiliated Qingyuan Hospital of Guangzhou Medical University, Qingyuan People's Hospital Qingyuan Guangdong China

**Keywords:** DL‐3‐n‐butylphthalide, DRP1, mitochondrial dysfunction, NRF2, OPA1, oxidative stress, prion diseases

## Abstract

**Background:**

Prion diseases are fatal neurodegenerative diseases caused by misfolded prion protein. DL‐3‐n‐butylphthalide (NBP), a synthetic agent derived from celery seeds, exhibits neuroprotective effects in multiple neurological disorders. However, its effects against prion peptide‐induced neurotoxicity remain unclear.

**Methods:**

A PrP^106‐126^‐induced neurotoxicity model was established in N2a cells to evaluate the effects of NBP. Apoptosis, oxidative stress, mitochondrial function, mitochondrial dynamics, and respiratory chain integrity were assessed following NBP pretreatment. Mechanistic causality was examined using OPA1 knockdown, DRP1 overexpression, NRF2 inhibition, and NRF2 overexpression. Key findings were further validated in human iPSC‐derived neurons.

**Results:**

NBP attenuated PrP^106‐126^‐induced neuronal apoptosis, decreasing cytochrome *c* release and caspase 3 cleavage. NBP also alleviated oxidative stress by lowering ROS and MDA levels, restoring T‐AOC and SOD activity, and increasing NRF2/HO‐1 signaling. In parallel, NBP preserved mitochondrial integrity and bioenergetics by maintaining MMP, ATP production, OCR, and mtDNA content, while sustaining respiratory chain complex expression and activity. NBP further normalized mitochondrial dynamics, restoring OPA1 levels and reducing DRP1 enrichment in mitochondrial fractions. Functionally, OPA1 knockdown, DRP1 overexpression, or NRF2 inhibition abolished NBP‐mediated protection, whereas NRF2 overexpression recapitulated key protective effects and normalized OPA1/DRP1‐related markers. Consistent protective trends were observed in human iPSC‐derived neurons.

**Conclusion:**

NBP mitigates PrP^106‐126^‐induced neurotoxicity by engaging NRF2‐dependent antioxidant signaling and preserving mitochondrial homeostasis, with associated normalization of OPA1/DRP1‐related mitochondrial dynamics. These findings support further evaluation of NBP in prion disease‐relevant models.

## Graphical abstract

NBP counteracts PrP^106‐126^‐induced neurotoxicity by activating NRF2 and restoring OPA1/DRP1‐mediated mitochondrial dynamics. It suppresses oxidative stress and preserves mitochondrial function and bioenergetics. These actions support NBP as a promising therapeutic candidate for prion‐related neurodegeneration.

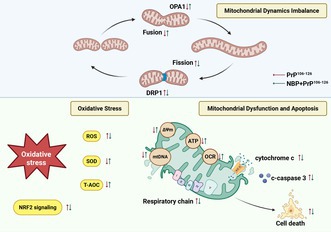

## 1 Introduction

Prion diseases are fatal neurodegenerative diseases in both humans and animals, including cattle, sheep, deer, camels, and cats [1]. They are caused by the misfolding of the host‐encoded cellular prion protein (PrP^C^) into its pathogenic isoform (PrP^Sc^), which aggregates in the brain and promotes neurodegeneration [2]. Owing to the unusual protein‐only nature of the pathogen, together with the lack of reliable early diagnostic biomarkers, therapeutic development for prion diseases remains extremely limited, and no interventions are currently available to effectively halt or reverse disease progression [3].

Although the molecular underpinnings of neuronal degeneration are still not fully elucidated, growing evidence points to redox imbalance and mitochondrial impairment as common pathological signatures in prion‐related neurotoxicity and other neurodegenerative disorders, including Alzheimer's disease (AD) and Parkinson's disease (PD) [4, 5, 6]. In our previous work, we found that prion peptide‐associated neurotoxicity is closely linked to mitochondrial impairment, including mitochondrial membrane potential (MMP) depolarization, ATP depletion, and dysregulated mitochondrial dynamics [7, 8]. These alterations are governed in part by the coordinated actions of dynamin‐related protein 1 (DRP1) and optic atrophy 1 (OPA1). An imbalance in the OPA1/DRP1 axis can promote excessive mitochondrial fragmentation, bioenergetic failure, and mitochondria‐dependent apoptotic signaling [7, 8, 9]. In parallel, prion peptides elevate ROS production and weaken antioxidant defenses, thereby exacerbating oxidative damage and neuronal loss [9]. Collectively, restoring mitochondrial dynamics and redox homeostasis may provide therapeutic benefit in prion‐related neurodegeneration.

Recent studies have highlighted DL‐3‐n‐butylphthalide (NBP) as a promising neuroprotective small molecule. NBP, a synthetic agent derived from celery seeds, is clinically approved in China as a treatment for ischemic stroke [10, 11, 12]. Clinically, NBP has been associated with improved neurological outcomes in ischemic stroke, with proposed mechanisms involving modulation of neurovascular function and cerebral hemodynamics [13]. Beyond stroke, accumulating studies in AD, PD, and other neurological disorder models suggest that NBP confers neuroprotection by attenuating oxidative stress, preserving mitochondrial bioenergetics, and reducing apoptosis [14, 15, 16, 17, 18, 19, 20]. Mechanistically, NBP has been reported to engage antioxidant defense pathways, particularly the NRF2/HO‐1 axis [19, 21, 22, 23]. Notably, to our knowledge, evidence supporting NBP in prion diseases remains limited, and it has not been clinically evaluated in prion patients. Therefore, whether NRF2‐dependent antioxidant signaling links NBP to restoration of OPA1/DRP1‐mediated mitochondrial dynamics and mitochondrial function under prion peptide‐associated neurotoxicity remains to be defined.

Due to the high infectivity and unique biochemical characteristics of prions, mechanistic studies of prion‐associated neurotoxicity are often initiated in vitro systems under standard biosafety conditions. To model prion protein‐induced neurotoxicity, we used the synthetic prion protein fragment PrP106‐126 (PrP^106‐126^), a widely used paradigm that recapitulates several biochemical properties and neurotoxic effects associated with PrP^Sc^ [24, 25]. Importantly, this model has been broadly applied to interrogate mitochondrial dysfunction, oxidative stress, and apoptosis in neuronal cells and to evaluate candidate neuroprotective interventions in a controlled setting [7, 8, 9, 26, 27, 28]. In this study, we investigated whether NBP mitigates PrP^106‐126^‐induced neurotoxicity by restoring mitochondrial function and redox balance, with a focus on NRF2/HO‐1 signaling and the OPA1/DRP1 axis governing mitochondrial dynamics. Mechanistic causality was further assessed using NRF2 inhibition (ML385) and NRF2 overexpression, together with OPA1 knockdown and DRP1 overexpression. In addition, key findings were validated in human iPSC‐derived neurons to strengthen translational relevance.

## 2 Materials and Methods

### 2.1 Cell Culture

The N2a mouse neuroblastoma cell line (ATCC, CCL‐131) was maintained in high‐glucose DMEM supplemented with 10% FBS and 1% penicillin‐streptomycin at 37°C under a humidified 5% CO_2_ atmosphere.

### 2.2 Human iPSC‐Derived Neuronal Differentiation

DYR0100 human iPSCs (ATCC, AC100378) were dissociated with Accutase and plated onto Matrigel‐precoated 6‐well plates at a density of 1.5 × 10^4^ cells/well in mTeSR1 medium supplemented with 10 μM Y‐27632 on day 0. Cells were maintained at 37°C in a humidified incubator. On day 1, rtTA and TetO‐hNgn2‐P2A‐PuroR lentiviruses were added to fresh mTeSR1 medium containing 10 μg/mL polybrene at 5 μL/well. On day 2, the medium was replaced with N2 medium (DMEM/F12 supplemented with 1× NEAA and 1× N2; prepared from 500 mL DMEM/F12, 5 mL NEAA, and 5 mL N2) containing 20 μg/L human BDNF, 20 μg/L human NT‐3, 0.2 mg/L mouse laminin, and 2 mg/L doxycycline. Doxycycline was maintained in the culture medium until the end of the experiment. On day 3, cells were cultured in fresh N2 medium supplemented with 1 mg/L puromycin and 2 mg/L doxycycline for 24 h. On day 4, selection was continued in N2 medium containing 4 mg/L puromycin and 2 mg/L doxycycline for another 24 h. On day 5, the medium was changed to B27/GlutaMAX neuronal medium (Neurobasal supplemented with GlutaMAX and B27; prepared from 500 mL Neurobasal, 5 mL GlutaMAX, and 10 mL B27) containing 2 mg/L doxycycline, 2 mg/L cytosine arabinoside, 20 μg/L BDNF, and 20 μg/L NT‐3. Cells were maintained in this medium until harvest on day 8.

### 2.3 Peptide and Compound Preparation

PrP peptide PrP^106‐126^ (sequence: KTNMKHMAGAAAAGAVVGGLG, > 98% purity; Sangon Biotech, China) was reconstituted in PBS (Solarbio, P1020, USA) at 1 mM. To induce aggregation, the solution was shaken for 24 h at 4°C. N2a cells underwent a 24 h exposure to PrP^106‐126^ (100 μM).

DL‐3‐n‐butylphthalide (NBP; MedChemExpress, HY‐B0647, USA) was dissolved in DMSO (Sigma‐Aldrich, D2650, USA) to yield a 10 mM stock solution. Cells underwent a 24 h pretreatment with NBP (10 mM) before PrP^106‐126^ exposure.

ML385 (MedChemExpress, HY‐100523, USA) was prepared in DMSO (5 mM). N2a cells underwent a 12 h pretreatment with ML385 (5 mM) before PrP^106‐126^ exposure.

### 2.4 Transfection

For mitochondrial visualization, N2a cells were transfected with a Mito‐GFP plasmid (Clontech, USA) using Lipofectamine 3000 (Invitrogen, L3000015, USA) for 48 h. To manipulate mitochondrial dynamics, OPA1 knockdown was achieved using siRNA (5′‐GUUAUCAGUCUGAGCCAGGTT‐3′; Synbio Technologies, China) [7], DRP1 overexpression was induced by transfection with the PCMV‐HA‐DRP1 plasmid (Synbio Technologies, China) [8], and NRF2 overexpression was induced by transfection with the PCMV‐HA‐NRF2 plasmid (Synbio Technologies, China).

### 2.5 Cell Viability and Apoptosis Detection

Cell Counting Kit‐8 (CCK‐8; Beyotime, C0038, China) was used to assess cell viability. CCK‐8 reagent was added to N2a cells and cultured for 2 h, followed by absorbance measurement at 450 nm using a Cytation microplate reader.

One‐Step TUNEL Apoptosis Assay Kit (Beyotime, C1086, China) was applied to evaluate apoptosis. Cells were cultured with TUNEL working solution at 37°C for 1 h, and fluorescence signals were captured with an Olympus confocal imaging system.

### 2.6 Subcellular Fractionation and Western Blotting

Mitochondrial and cytoplasmic fractions were prepared, and Western blotting was performed as previously described [9]. Membranes were incubated with primary antibodies against: Cytochrome *c* (Abcam, ab133504, USA), cleaved Caspase 3 (Cell Signaling Technology, 9661, USA), NRF2 (Proteintech, 16,396–1‐AP, China), HO‐1 (Proteintech, 81,281–1‐RR, China), NDUFB8 (Proteintech, 14,794–1‐AP, China), SDHB (Proteintech, 67,600–1‐Ig, China), UQCRC1 (Proteintech, 21,705–1‐AP, China), MTCO2 (Proteintech, 55,070–1‐AP, China), ATP5A1 (Proteintech, 14,676–1‐AP, China), OPA1 (Cell Signaling Technology, 80,471, USA), DRP1 (Cell Signaling Technology, 8570, USA), VDAC (Cell Signaling Technology, 4661, USA), β‐Tubulin (Proteintech, 10,094–1‐AP, China), and GAPDH (Proteintech, 60,004–1‐AP, China).

### 2.7 Oxidative Stress Detection

The Reactive Oxygen Species Assay Kit (Beyotime, S0033, China) was applied to measure ROS, the Total Antioxidant Capacity Assay Kit (Rapid ABTS method) (Beyotime, S0121, China) for total antioxidant capacity (T‐AOC), the Lipid Peroxidation MDA Assay Kit (Beyotime, S0131S, China) for malondialdehyde (MDA), and the Total Superoxide Dismutase Assay Kit (WST‐8 method) (Beyotime, S0101S, China) for superoxide dismutase (SOD). ROS fluorescence was observed using an Olympus confocal imaging system or a Cytation microplate reader, while absorbance for T‐AOC, MDA, and SOD was determined at the corresponding wavelengths with a Cytation microplate reader.

### 2.8 Transmission Electron Microscopy (TEM)

Treated N2a cells were processed for TEM as previously described [7]. Images were captured with a Tecnai G2 Spirit TEM at 120 kV.

### 2.9 Mitochondrial Function Detection

The Tetramethylrhodamine Ethyl Ester (TMRE) Assay Kit (Beyotime, C2001, China) was used to assess MMP, and fluorescence was observed using an Olympus confocal imaging system or a Cytation microplate reader. The Enhanced ATP Assay Kit (Beyotime, S0027, China) was used to measure intracellular ATP levels, and luminescence was recorded with a Cytation microplate reader. The Oxygen Consumption Rate Assay Kit (Elabscience, E‐BC‐F068, China) was used to determine the oxygen consumption rate (OCR), and absorbance was recorded with a Cytation microplate reader.

### 2.10 Mitochondrial DNA Copy Number Quantification

The Universal Genomic DNA Extraction Kit (CWBIO, CW2298S, China) was used to isolate total DNA from N2a cells, and DNA concentration was determined with a NanoDrop 2000 spectrophotometer. The mitochondrial DNA (mtDNA) copy number was quantified by qPCR using the SYBR Green Master Mix (Vazyme, Q141‐02, China) on a ViiA7 Fast Real‐Time PCR System. Primer sequences were as follows: mtDNA forward 5′‐CCTATCACCCTTGCCATCAT‐3′, reverse 5′‐GAGGCTGTTGCTTGTGTGAC‐3′; nDNA forward 5′‐ATGGAAAGCCTGCCATCATG‐3′, reverse 5′‐TCCTTGTTGTTCAGCATCAC‐3′. Relative mtDNA levels were calculated using the 2 − ΔΔCT method.

### 2.11 Mitochondrial Respiratory Complex Activity Assays

The Complex I Activity Assay Kit (Solarbio, BC0510, China), Complex II Activity Assay Kit (Solarbio, BC3230, China), Complex III Activity Assay Kit (Solarbio, BC3240, China), and Complex IV Activity Assay Kit (Solarbio, BC0940, China) were used to assess the enzymatic activities of respiratory chain complexes I–IV. Absorbance was recorded with a Cytation microplate reader.

### 2.12 Statistical Analysis

Quantitative results are shown as mean ± SD. Statistical evaluation was carried out with GraphPad Prism 9. Student's *t*‐test was applied for two‐group comparisons, and ANOVA with Tukey's correction was used for multiple comparisons. All experiments were performed in at least three independent biological replicates. Significance was set at *p* < 0.05.

## 3 Results

### 3.1 NBP Suppresses PrP^106^ ^−126^‐Induced Apoptosis in N2a Cells

**FIGURE 1:**
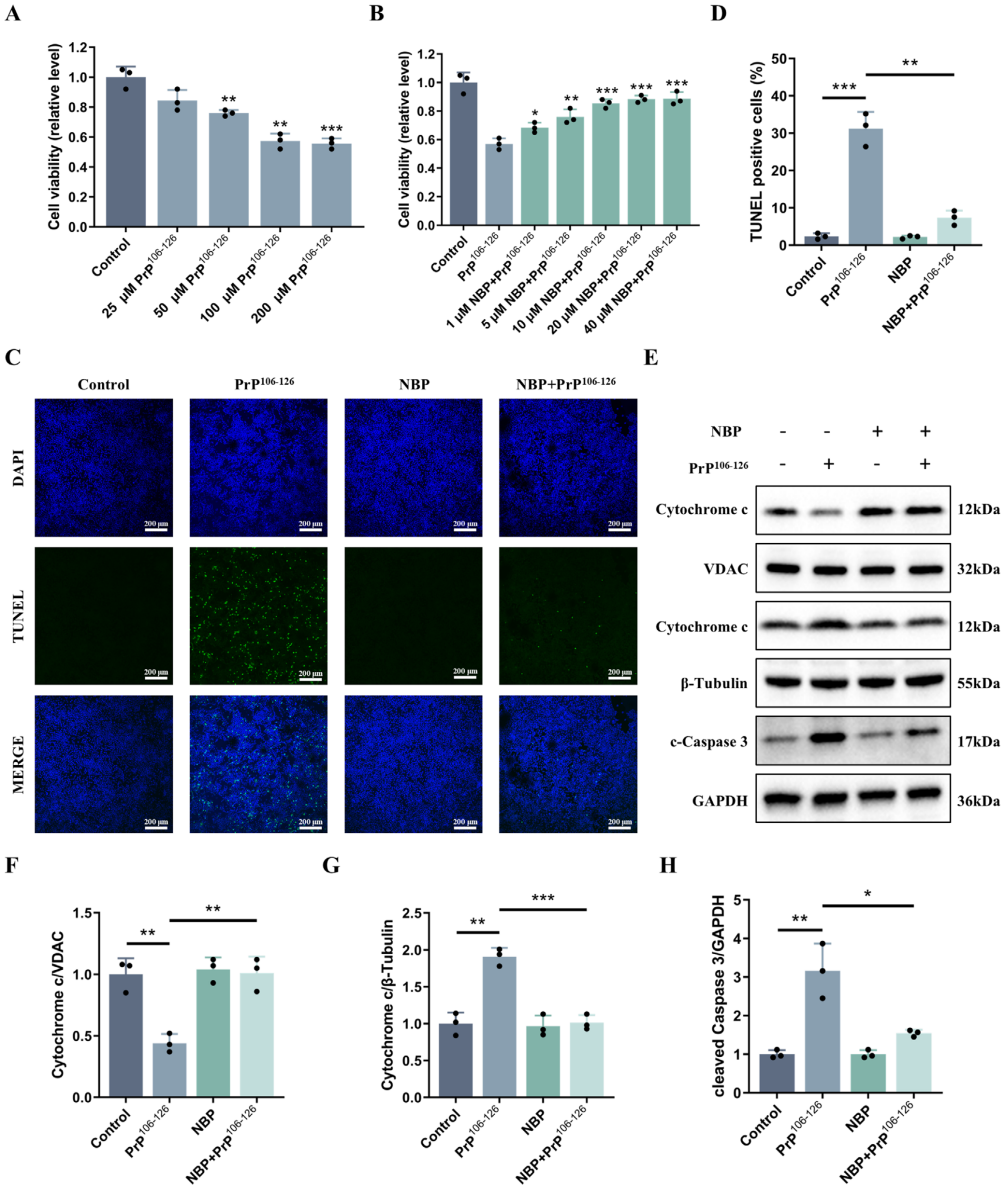
NBP suppresses PrP^106−126^‐induced apoptosis in N2a cells (A, B) Cell viability (CCK‐8 assay). (C, D) Apoptosis (TUNEL staining, scale bar = 200 μm). (E–H) Cytochrome *c* (mitochondrial and cytosolic fractions) and cleaved caspase 3 protein expression (western blot). Data are presented as mean ± SD (*n* = 3). *p < 0.05, **p < 0.01, ***p < 0.001.

To establish a cellular model of prion toxicity, N2a cells underwent 24 h exposure to PrP^106‐126^ at graded doses (25–200 μM). Cell viability progressively declined with increasing peptide levels, and exposure to 100 μM resulted in nearly maximal suppression comparable to 200 μM (Figure 1A). Thus, 100 μM was chosen in later assays as the working concentration to model prion‐induced cytotoxicity.

We next examined whether NBP could counteract this injury. Before peptide treatment, cells were subjected to a 24 h pretreatment with NBP (1–40 μM) at graded concentrations. A concentration‐dependent improvement in viability was observed, and 10 μM NBP provided protection equivalent to higher doses (Figure 1B). Accordingly, 10 μM was selected for mechanistic analyses.

The anti‐apoptotic effect of NBP was further validated by TUNEL staining. PrP^106‐126^ markedly increased DNA fragmentation after 24 h, whereas NBP pretreatment substantially lowered the proportion of apoptotic cells (Figure 1C,D).

To delineate the molecular basis of this effect, we focused on the mitochondrial apoptotic pathway. PrP^106−126^ exposure promoted cytosolic translocation of cytochrome *c* and markedly enhanced caspase 3 activity (Figure 1E–H). Pretreatment with NBP restrained cytochrome *c* redistribution and attenuated caspase 3 cleavage (Figure 1E–H).

Overall, these results suggest that NBP preserves neuronal survival under prion peptide stress by restraining mitochondria‐dependent apoptotic signaling.

### 3.2 NBP Reduces PrP^106−^ ^126^‐Induced Oxidative Stress in N2a Cells

**FIGURE 2:**
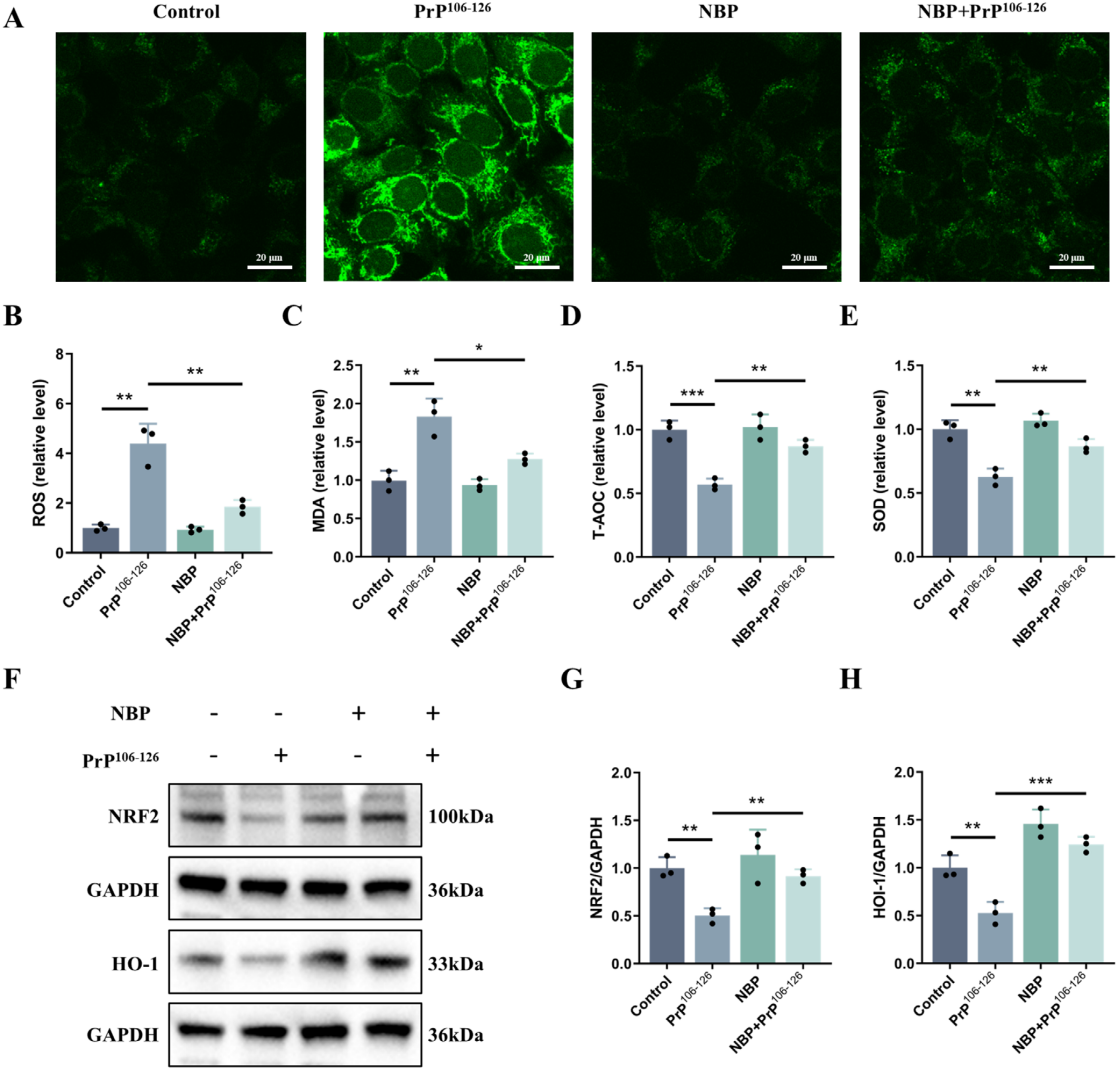
NBP reduces PrP^106−126^‐induced oxidative stress in N2a cells (A, B) ROS (DCFH‐DA staining, scale bar = 20 μm). (C) MDA content. (D) T‐AOC level. (E) SOD activity. (F–H) NRF2 and HO‐1 protein expression (western blot). Data are presented as mean ± SD (*n* = 3). *p < 0.05, **p < 0.01, ***p < 0.001.

Because oxidative stress is a key contributor to PrP^106‐126^‐induced neurotoxicity, we next examined whether NBP exerts antioxidant effects. Measurement of intracellular ROS revealed a pronounced increase following PrP^106‐126^ exposure, whereas pretreatment with NBP significantly suppressed ROS accumulation (Figure 2A,B). PrP^106−126^ also elevated MDA levels and reduced both T‐AOC and SOD activity, indicating enhanced lipid peroxidation and impaired antioxidant defenses (Figure 2C–E). NBP pretreatment largely reversed these alterations, restoring redox homeostasis.

To gain mechanistic insight, we examined NRF2 signaling, a central regulator of oxidative defense. Western blot analysis revealed that PrP^106−126^ markedly reduced the expression of NRF2 and HO‐1, whereas NBP pretreatment restored their levels (Figure 2F–H).

In summary, these data indicate that NBP counteracts oxidative stress through the NRF2 pathway, thereby re‐establishing antioxidant balance.

### 3.3 NBP Alleviates PrP^106−^ ^126^‐Induced Mitochondrial Dysfunction in N2a Cells

**FIGURE 3:**
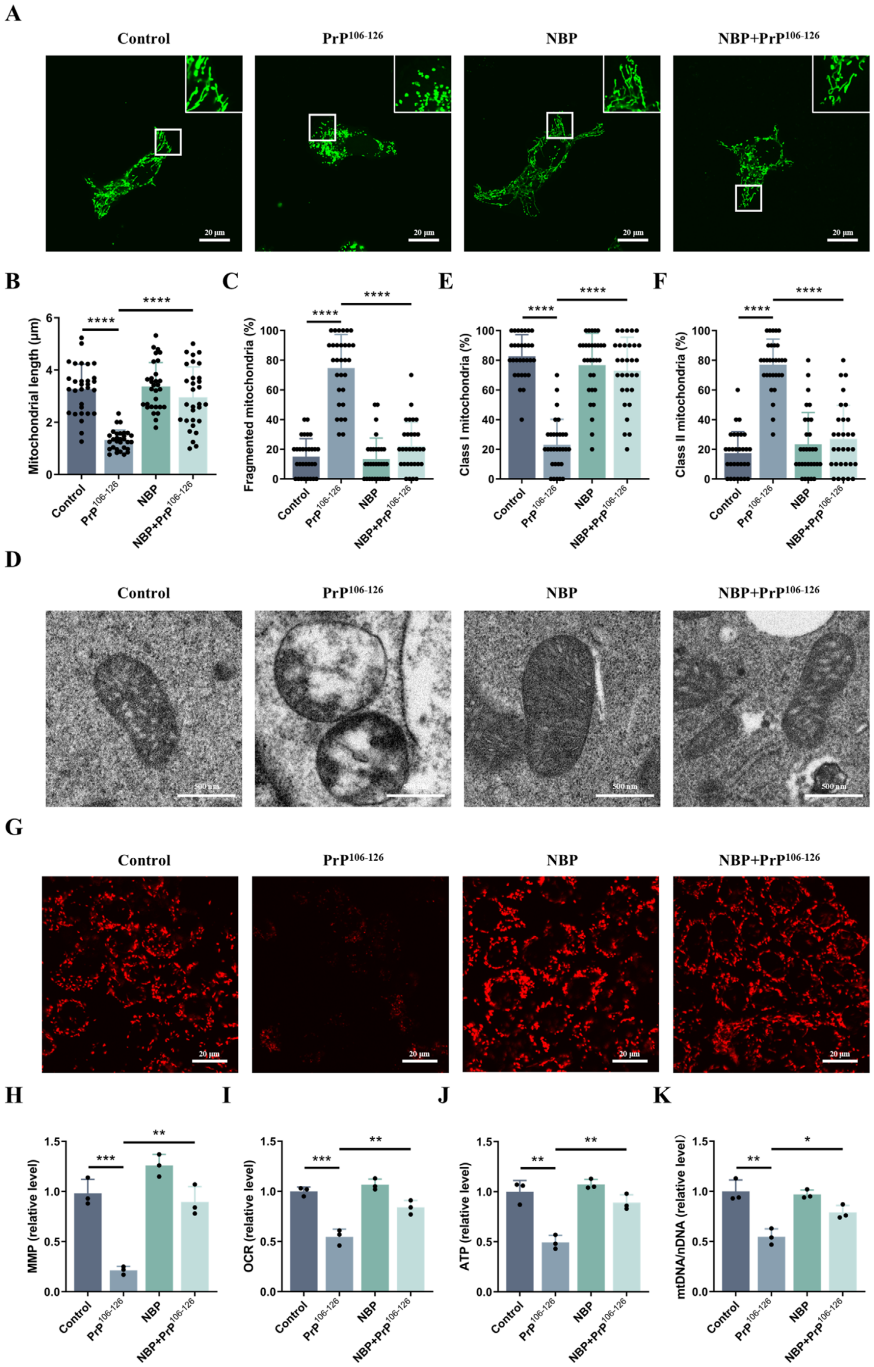
NBP alleviates PrP^106−126^‐induced mitochondrial dysfunction in N2a cells (A) Mitochondrial network morphology (confocal microscopy, scale bar = 20 μm). (B) Mitochondrial length. (C) Percentage of fragmented mitochondria. (D) Mitochondrial ultrastructure (TEM, scale bar = 500 nm). (E, F) Quantification of mitochondrial cristae number. Mitochondria with > 4 discernible cristae were classified as Class I, whereas those with ≤ 4 cristae were classified as Class II. (G, H) MMP (TMRE staining, scale bar = 20 μm). (I) OCR. (J) Intracellular ATP. (K) mtDNA/nDNA ratio (real‐time PCR). Data are presented as mean ± SD (*n* = 3). *p < 0.05, **p < 0.01, ***p < 0.001, ****p < 0.0001.

Given that mitochondrial impairment is a hallmark of PrP^106−126^‐induced neurotoxicity, we next investigated whether NBP preserved mitochondrial structural and functional integrity. Confocal microscopy revealed pronounced mitochondrial fragmentation in PrP^106−126^‐treated cells, characterized by shortened and punctate mitochondria (Figure 3A). Quantitative analysis confirmed a significant decrease in mitochondrial length and an increased percentage of fragmented mitochondria (Figure 3B,C). In contrast, NBP pretreatment largely preserved an elongated and interconnected mitochondrial network and partially restored these metrics (Figure 3A–C).

TEM further showed that PrP^106−126^ caused severe ultrastructural abnormalities, characterized by collapsed cristae (Figure 3D). Cristae remodeling was quantified by classifying mitochondria according to cristae number: mitochondria with > 4 cristae were defined as Class I, whereas those with ≤ 4 cristae were defined as Class II. PrP^106−126^ exposure significantly increased the proportion of Class II mitochondria with a corresponding decrease in Class I mitochondria, indicating severe cristae disruption (Figure 3E,F). Notably, NBP pretreatment attenuated these ultrastructural abnormalities, as reflected by a shift toward a higher proportion of Class I mitochondria (Figure 3D–F).

Consistent with these structural findings, functional impairment was also observed. PrP^106−126^ significantly reduced MMP, OCR, and intracellular ATP levels, all of which were restored by NBP pretreatment (Figure 3G–J). To further assess mitochondrial integrity, we measured mtDNA copy number. PrP^106‐126^ exposure markedly reduced mtDNA levels, whereas NBP pretreatment effectively reversed this decline (Figure 3K).

Together, these results demonstrate that NBP preserves mitochondrial integrity under prion peptide stress by maintaining morphology, bioenergetic function, and mtDNA content.

### 3.4 NBP Ameliorates PrP^106−^ ^126^‐Induced Respiratory Chain Impairment

**FIGURE 4:**
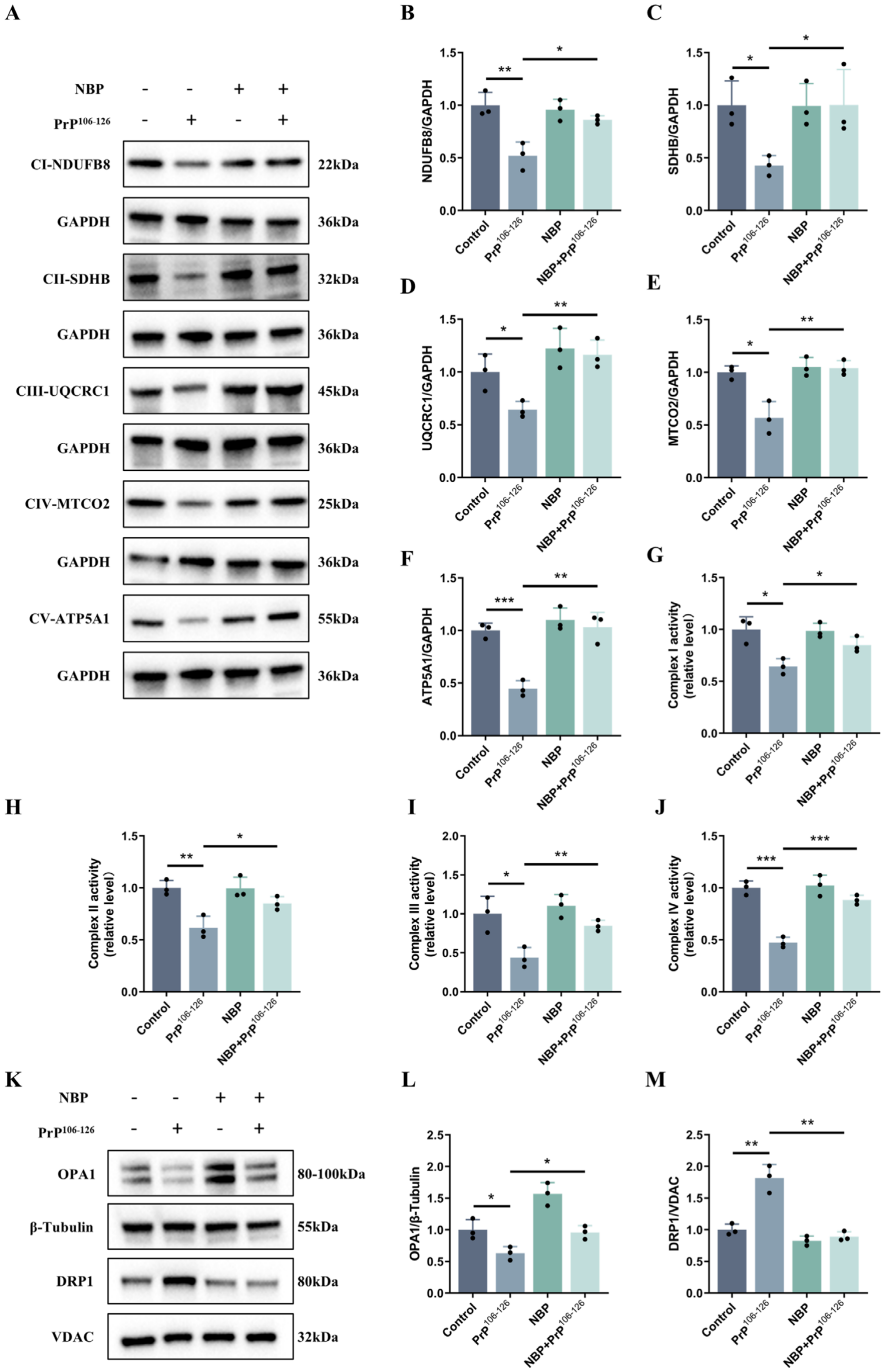
NBP ameliorates PrP^106−126^‐induced impairments in respiratory chain function and mitochondrial dynamics. (A–F) CI‐NDUFB8, CII‐SDHB, CIII‐UQCRC1, CIV‐MTCO2, and CV‐ATP5A1 protein expression (western blot). (G–J) Activities of respiratory chain complexes I–IV. (K–M) Total OPA1 and mitochondrial DRP1 protein expression (western blot). Data are presented as mean ± SD (*n* = 3). *p < 0.05, **p < 0.01, ***p < 0.001.

We further examined whether NBP affected the expression and activity of mitochondrial respiratory chain complexes. Western blotting revealed that PrP^106−126^ markedly reduced the protein levels of representative subunits from complexes I–V, including NDUFB8 of complex I, SDHB of complex II, UQCRC1 of complex III, MTCO2 of complex IV, and ATP5A1 of complex V, indicating broad suppression of respiratory chain components. In contrast, NBP pretreatment preserved the expression of these subunits (Figure 4A–F).

Consistent with these findings, complexes I–IV exhibited significantly diminished activities in response to PrP^106−126^, whereas NBP pretreatment restored their activities toward control levels (Figure 4G–J).

In summary, these results demonstrate that NBP sustains mitochondrial bioenergetics under prion peptide stress by maintaining respiratory chain integrity.

### 3.5 NBP Mitigates Mitochondrial Dynamics Through Regulation of OPA1 and DRP1

As disturbed mitochondrial dynamics are increasingly recognized in prion pathology, we next assessed whether NBP could modulate the balance between fusion and fission. Western blot analysis demonstrated that PrP^106−126^ exposure led to a reduction in total cellular OPA1 and a concomitant increase in mitochondrial DRP1, consistent with a shift toward excessive fission (Figure 4K–M). Pretreatment with NBP effectively counteracted these changes, restoring OPA1 levels and reducing mitochondrial DRP1 enrichment (Figure 4K–M).

These findings indicate that NBP helps maintain mitochondrial dynamics under prion peptide stress by regulating OPA1 levels and DRP1 distribution.

### 3.6 OPA1 Knockdown or DRP1 Overexpression Abolishes NBP‐Mediated Neuroprotection Against PrP^106−^ ^126^

**FIGURE 5:**
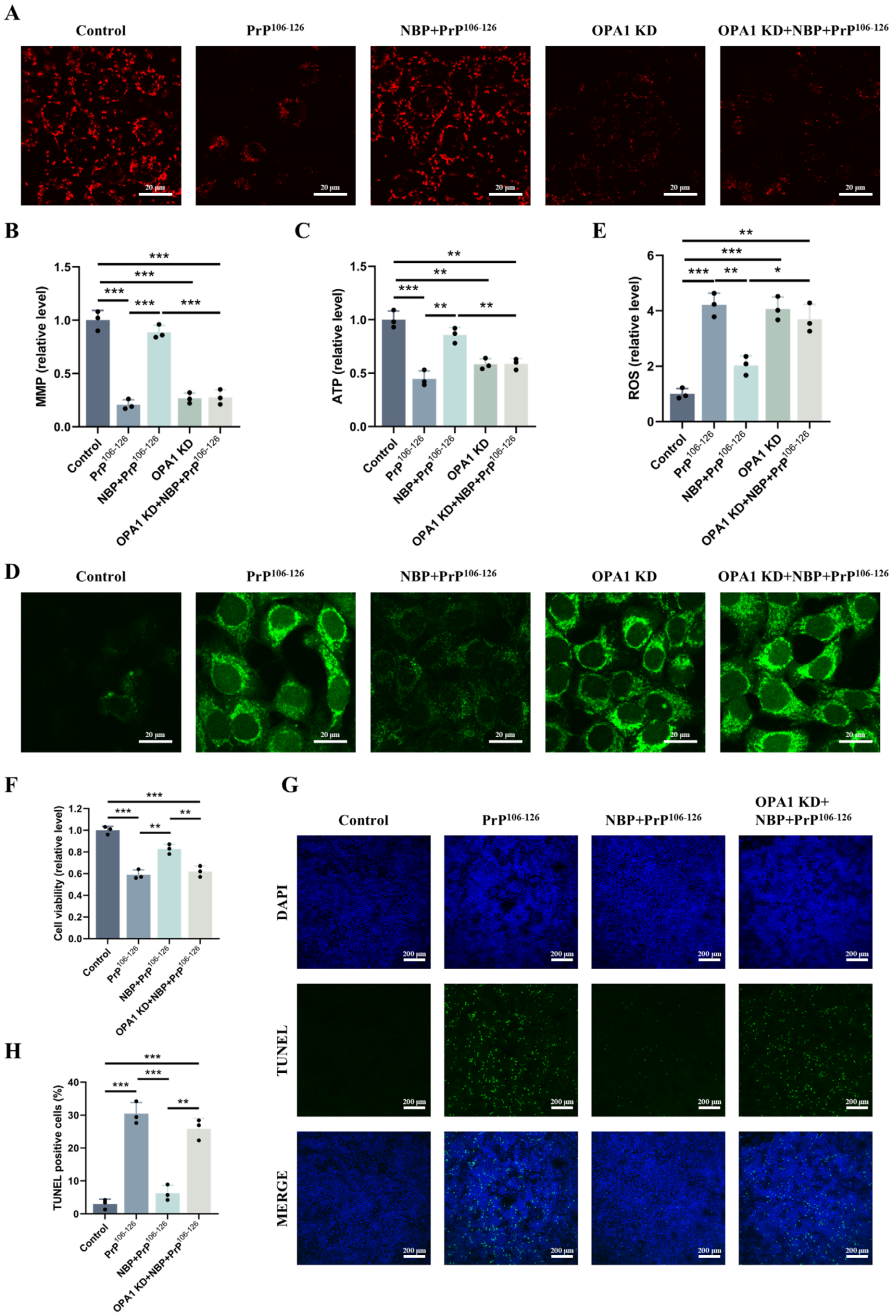
OPA1 knockdown abolishes NBP‐mediated neuroprotection against PrP^106−126^ (A, B) MMP (TMRE staining, scale bar = 20 μm). (C) Intracellular ATP. (D, E) ROS (DCFH‐DA staining, scale bar = 20 μm). (F) Cell viability (CCK‐8 assay). (G, H) Apoptosis (TUNEL staining, scale bar = 200 μm). Data are presented as mean ± SD (*n* = 3). *p < 0.05, **p < 0.01, ***p < 0.001.

To evaluate the role of mitochondrial dynamics in NBP‐mediated protection, we first silenced OPA1 expression in N2a cells. OPA1 knockdown reduced MMP and ATP levels while increasing ROS production, mimicking the effects of PrP^106−126^. Importantly, NBP pretreatment failed to restore MMP, ATP, or ROS levels in OPA1‐deficient cells, and the improvements in cell viability and apoptosis were abolished (Figure 5A–H). Complementing these findings, DRP1 overexpression, which promotes excessive mitochondrial fission, also resulted in decreased MMP and ATP and elevated ROS accumulation. Likewise, DRP1 overexpression prevented NBP from improving mitochondrial function, oxidative homeostasis, cell viability, or apoptosis following PrP^106−126^ exposure (Figure S1A–H).

Collectively, these results demonstrate that restoring mitochondrial dynamics—via OPA1‐mediated fusion and suppression of DRP1‐driven fission—is essential for the neuroprotective actions of NBP.

### 3.7 NBP Exerts Neuroprotection Against PrP^106^ ^−126^ via NRF2 Pathway

**FIGURE 6:**
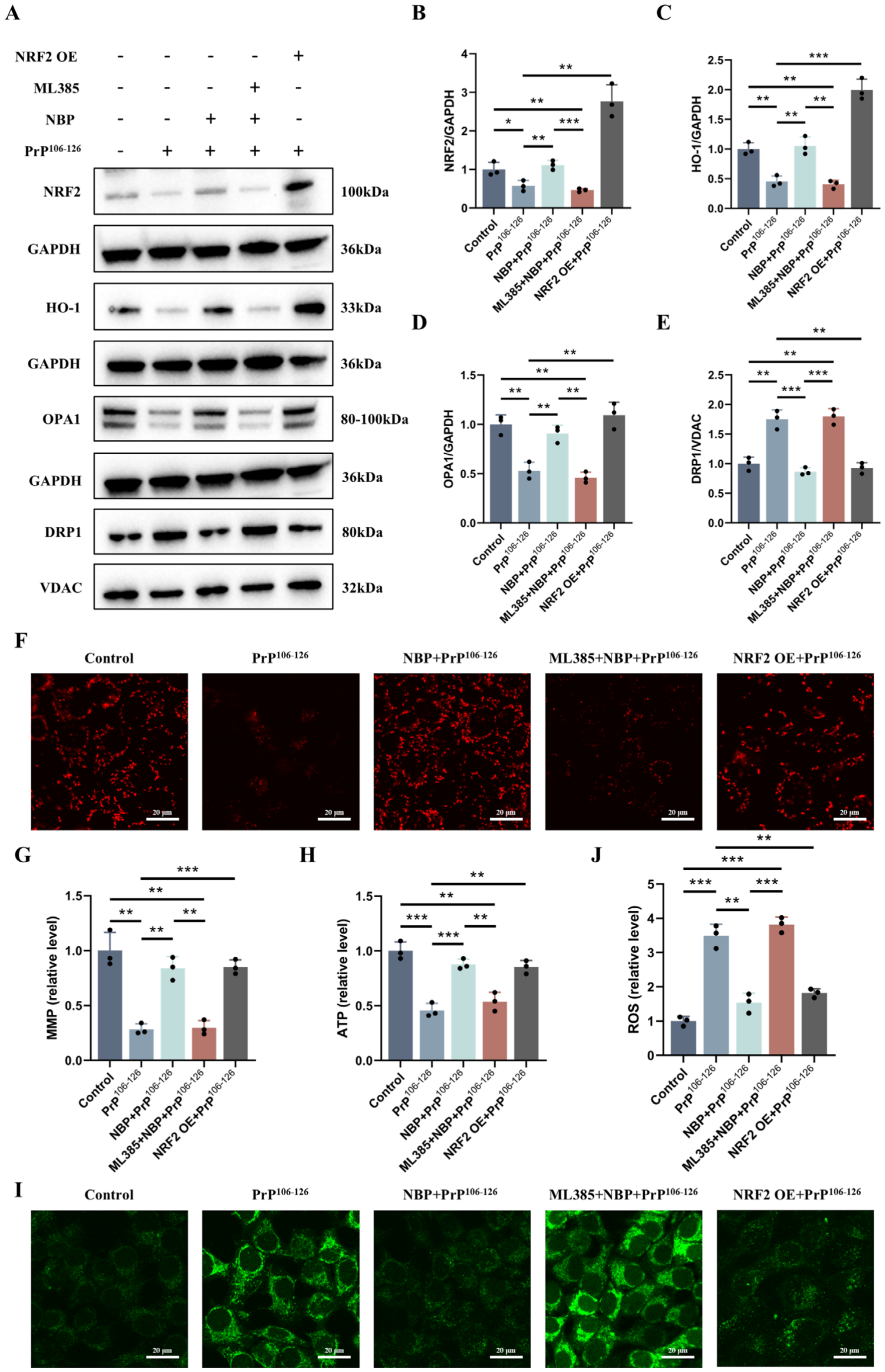
NBP exerts neuroprotection against PrP^106−126^ via NRF2 pathway (A–E) NRF2, HO‐1, total OPA1, and mitochondrial DRP1 protein expression (western blot). (F, G) MMP (TMRE staining, scale bar = 20 μm). (H) Intracellular ATP. (I, J) ROS (DCFH‐DA staining, scale bar = 20 μm). Data are presented as mean ± SD (*n* = 3). *p < 0.05, **p < 0.01, ***p < 0.001.

To assess the role of NRF2 in NBP‐mediated protection, NRF2 signaling was inhibited with ML385. ML385 markedly weakened the effects of NBP: it blunted HO‐1 induction and prevented restoration of OPA1 expression and reduction of DRP1 enrichment in mitochondrial fractions (Figure 6A–E). In parallel, ML385 abolished the functional benefits of NBP, such that MMP and ATP were no longer restored and the reduction of ROS was largely lost under PrP^106−126^ challenge (Figure 6F–J).

We next tested whether NRF2 activation is sufficient to confer protection by overexpressing NRF2 in PrP^106−126^‐treated N2a cells. NRF2 overexpression mimicked NBP treatment, inducing HO‐1, restoring the OPA1/DRP1 axis, improving MMP and ATP production, and reducing ROS accumulation (Figure 6A–J).

Together, these results indicate that NRF2 activation restores antioxidant signaling (HO‐1) and OPA1/DRP1‐associated mitochondrial dynamics, supporting NRF2 as an upstream mediator of NBP‐induced mitochondrial protection in the PrP^106−126^ neurotoxicity model.

### 3.8 NBP Attenuates PrP^106−^ ^126^‐Induced Mitochondrial Dysfunction and Oxidative Stress in Human iPSC‐Derived Neurons

Human iPSC‐derived neurons were used to further assess the translational relevance of NBP‐mediated neuroprotection (Figure S2A). PrP^106−126^ exposure significantly reduced MMP and ATP production and increased intracellular ROS levels in human neurons (Figure S2B–D). Notably, NBP treatment mitigated these alterations, restoring MMP and ATP levels and reducing ROS accumulation (Figure S2B–D). These trends were consistent with those observed in N2a cells.

Consistent with these functional changes, immunoblotting revealed that PrP^106−126^ exposure decreased NRF2 and HO‐1 expression in human neurons, accompanied by reduced OPA1 levels and increased mitochondrial enrichment of DRP1 (Figure S2E–I). NBP treatment largely reversed these effects, restoring NRF2/HO‐1 signaling, recovering OPA1 expression, and attenuating DRP1 recruitment to mitochondria (Figure S2E–I).

Overall, these results extend our observations to a human neuronal context, supporting the translational relevance of NBP‐mediated protection against PrP^106−126^‐induced mitochondrial and redox disturbances, in association with NRF2/HO‐1 activation and improved OPA1/DRP1‐related mitochondrial dynamics.

## 4 Discussion

Prion diseases are characterized by rapidly progressive neurodegeneration involving oxidative damage, mitochondrial impairment, and neuronal cell death. In the present study, we demonstrate that NBP effectively protects neuronal cells against PrP^106−126^‐induced neurotoxicity by concurrently mitigating oxidative stress and preserving mitochondrial integrity. Mechanistically, NBP restores NRF2/HO‐1 antioxidant signaling, which contributes to normalization of OPA1/DRP1‐related mitochondrial dynamics, thereby reducing mitochondrial fragmentation, sustaining respiratory chain function, and attenuating mitochondria‐dependent apoptotic signaling. These conclusions are supported by rescue experiments showing that OPA1 knockdown, DRP1 overexpression, or NRF2 inhibition each abolishes NBP's protective effects. Moreover, key findings were recapitulated in human iPSC‐derived neurons, strengthening their translational relevance.

NBP is clinically approved in China for the treatment of ischemic stroke, supporting its translational relevance as a neuroprotective agent [10, 11, 12]. Beyond its approved indication, accumulating evidence from diverse neurological disorder models suggests that NBP exerts protective effects by reinforcing antioxidant defenses, preserving mitochondrial bioenergetics/respiratory chain function, and attenuating mitochondria‐dependent cell death signaling. For example, in focal ischemia models, NBP prevents neuronal apoptosis and limits ROS accumulation by inhibiting mitochondrial apoptotic pathways and suppressing JNK/p38 signaling [29], whereas in an in vitro ischemic stroke model it enhances antioxidant defenses, upregulates NRF2/HO‐1 signaling, and preserves mitochondrial function and dynamics [30]. NBP has also been reported to improve mitochondrial function in cerebral ischemia/reperfusion injury through upregulation of cytochrome c oxidase 7c (Cox7c) [31] and to protect dopaminergic neurons in PD models by attenuating mitochondrial impairment and oxidative stress [18]. More recently, NBP has also been reported to reduce oxidative stress and protect mitochondria in traumatic brain injury and ferroptosis‐related neuronal injury models [32]. Notably, oxidative stress, mitochondrial dysfunction, and apoptosis are also central drivers of PrP^106−126^‐induced neurotoxicity. Consistent with this rationale, our results show that NBP pretreatment ameliorates PrP^106−126^‐induced neurotoxicity by reducing ROS and MDA levels, restoring T‐AOC and SOD activity, preserving MMP and ATP production, and attenuating cytochrome *c* release and caspase 3 cleavage in N2a cells. Importantly, our loss‐ and gain‐of‐function data provide causal evidence that NRF2 activity is an upstream mediator of this integrated response: NRF2 inhibition with ML385 blunted HO‐1 induction and abolished NBP's neuroprotective ability, whereas NRF2 overexpression recapitulated key aspects of NBP protection, improving redox balance and mitochondrial function under PrP^106−126^ challenge. Together, these findings strengthen the mechanistic linkage between NRF2 signaling, antioxidant defense, and mitochondrial preservation in prion peptide‐associated neurotoxicity. While NBP has been reported to activate NRF2 in other disease contexts [14, 19, 21, 22, 23, 33, 34, 35], our study extends this concept by providing causal evidence that NRF2 activation is sufficient to rescue antioxidant signaling and mitochondrial protection under PrP^106−126^ stress, with accompanying normalization of OPA1/DRP1‐related markers.

The importance of rebalancing OPA1/DRP1 was supported by rescue experiments showing that OPA1 knockdown or DRP1 overexpression abolished NBP's protective effects. NBP's ability to modulate mitochondrial dynamics has also been reported in other models, albeit with different mechanistic emphases: in cerebral ischemia, NBP inhibits DRP1 acetylation/phosphorylation [36]; in postoperative cognitive dysfunction, NBP activates NRF2/ARE to upregulate MFN1, MFN2, and DRP1 [37]; and in cerebral ischemia–reperfusion, NBP suppresses OMI/HTRA2 signaling and reduces OPA1 expression [38]. To our knowledge, the present study demonstrates that, in the context of PrP^106−126^‐induced neurotoxicity, NBP restores OPA1 expression and reduces mitochondrial DRP1 recruitment through an NRF2‐dependent mechanism, as NRF2 inhibition abrogated these effects and NRF2 overexpression mimicked them. Thus, while NBP engages diverse molecular pathways to preserve mitochondrial dynamics depending on the pathological context, its targeting of the NRF2‐OPA1/DRP1 axis represents a previously underappreciated mechanism in prion peptide‐associated neurotoxicity.

The observation that NBP similarly ameliorated PrP^106−126^‐induced mitochondrial dysfunction and oxidative stress in human iPSC‐derived neurons substantially increases the translational potential of our findings. Human neurons recapitulated the alterations in NRF2/HO‐1 signaling and OPA1/DRP1‐related markers, and NBP treatment exerted consistent protective effects, suggesting that the identified pathway is not merely a murine cell line phenomenon. Nevertheless, several limitations should be acknowledged. We used a PrP^106−126^‐induced in vitro neurotoxicity model, which enables mechanistic interrogation under standard biosafety conditions but does not recapitulate infectious prion replication/propagation or tissue‐level neuropathology. Although key findings were validated in human iPSC‐derived neurons, in vivo verification in certified prion disease models will be important for future translational development.

## 5 Conclusion

This study identifies NBP as a potent neuroprotective agent against PrP^106−126^‐induced neurotoxicity. By activating NRF2‐dependent antioxidant signaling and subsequently normalizing OPA1/DRP1‐related mitochondrial dynamics, NBP preserves mitochondrial bioenergetics, reduces oxidative damage, and limits mitochondria‐dependent apoptotic cell death. Given that NBP is already approved for clinical use in ischemic stroke in China, its existing clinical use supports its potential for repurposing, pending further validation in prion disease models. Our findings provide a rationale for further preclinical evaluation of NBP in animal models of prion infection when such studies become feasible.

## Author Contributions

W.W. conceived and designed the study. W.W., X.Z., and M.J. conducted the experiments. W.W. and N.M. revised and edited the manuscript. All authors approved the final manuscript.

## Funding

This work was supported by the China Postdoctoral Science Foundation, 2021M703235. Basic and Applied Basic Research Foundation of Guangdong Province, 2022A1515110669. National Natural Science Foundation of China, 82200355, 82470243. Research Fund of Qingyuan People's Hospital, 202201‐208.

## Ethics Statement

This study did not involve human participants, clinical data, or animal experiments. Therefore, ethical approval was not required.

## Conflicts of Interest

The authors declare no conflicts of interest.

## Supporting information


**Figure S1:** DRP1 overexpression negates the neuroprotective actions of NBP against PrP^106‐126^.
(A, B) MMP (TMRE staining, scale bar = 20 μm). (C) Intracellular ATP. (D, E) ROS (DCFH‐DA staining, scale bar = 20 μm). (F) Cell viability (CCK‐8 assay). (G, H) Apoptosis (TUNEL staining, scale bar = 200 μm). Data are presented as mean ± SD (*n* = 3). *p < 0.05, **p < 0.01, ***p < 0.001, ****p < 0.0001.

**Figure S2:** NBP attenuates PrP^106‐126^‐induced mitochondrial dysfunction and oxidative stress in human iPSC‐derived neurons.
(A) Schematic diagram and representative images of human iPSC‐derived neurons. (B) MMP (TMRE staining). (C) Intracellular ATP. (D) ROS (DCFH‐DA staining). (E‐I) NRF2, HO‐1, total OPA1, and mitochondrial DRP1 protein expression (western blot). Data are presented as mean ± SD (*n* = 3). *p < 0.05, **p < 0.01, ***p < 0.001.

## Data Availability

The data that support the findings of this study are available from the corresponding author upon reasonable request.
