# Supplementary figures and images for: DL‐3‐n‐Butylphthalide Protects Against PrP106 −126‐Induced Neurotoxicity Through NRF2 Signaling and OPA1/DRP1‐Mediated Mitochondrial Dynamics
Source: CNS Neurosci Ther. 2026 Jun 2;32(6):e70948. doi: 10.1002/cns.70948 (PMC13240468; doi:10.1002/cns.70948)

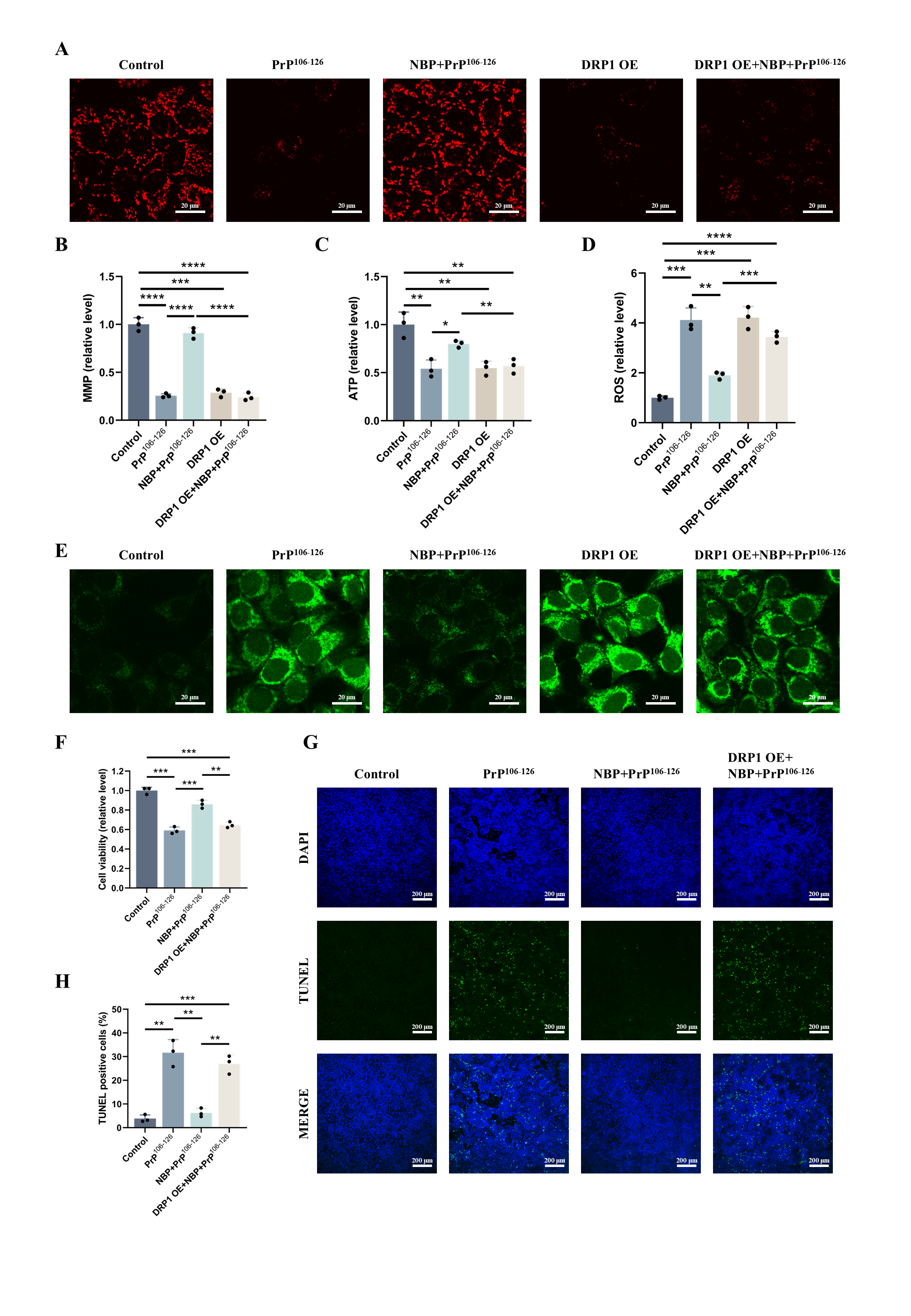

Supplement: Supplementary file 1 — Figure S1: DRP1 overexpression negates the neuroprotective actions of NBP against PrP106‐126. (A, B) MMP (TMRE staining, scale bar = 20 μm). (C) Intracellular ATP. (D, E) ROS (DCFH‐DA staining, scale bar = 20 μm). (F) Cell viability (CCK‐8 assay). (G, H) Apoptosis (TUNEL staining, scale bar = 200 μm). Data are presented as mean ± SD (n = 3). *p < 0.05, **p < 0.01, ***p < 0.001, ****p < 0.0001. [file CNS-32-e70948-s001.tif]

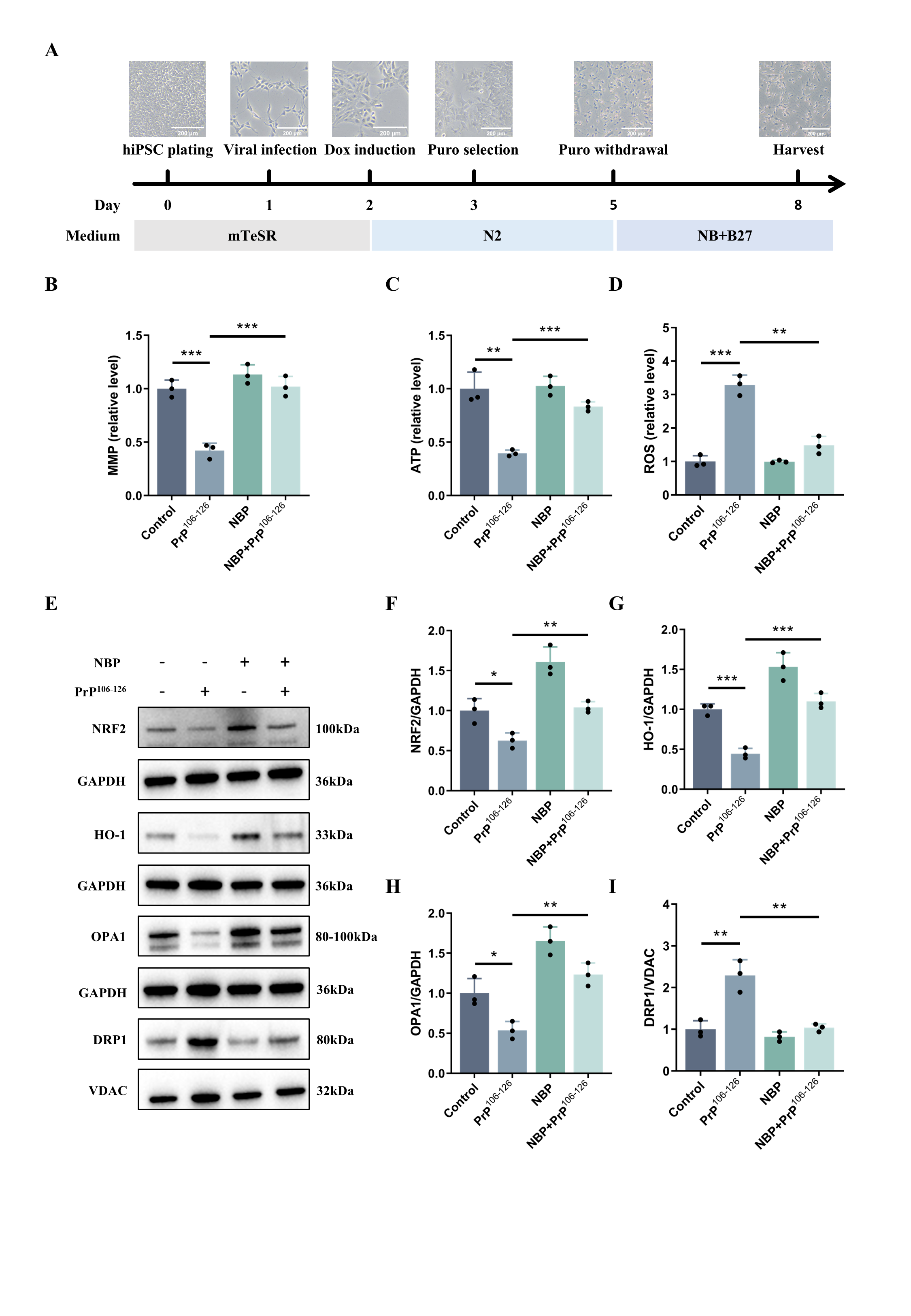

Supplement: Supplementary file 2 — Figure S2: NBP attenuates PrP106‐126‐induced mitochondrial dysfunction and oxidative stress in human iPSC‐derived neurons. (A) Schematic diagram and representative images of human iPSC‐derived neurons. (B) MMP (TMRE staining). (C) Intracellular ATP. (D) ROS (DCFH‐DA staining). (E‐I) NRF2, HO‐1, total OPA1, and mitochondrial DRP1 protein expression (western blot). Data are presented as mean ± SD (n = 3). *p < 0.05, **p < 0.01, ***p < 0.001. [file CNS-32-e70948-s002.tif]
